# C-type natriuretic peptide preserves central neurological function by maintaining blood-brain barrier integrity

**DOI:** 10.3389/fnmol.2022.991112

**Published:** 2022-10-04

**Authors:** Cristina Perez-Ternero, Patrick N. Pallier, Jordi L. Tremoleda, Alessio Delogu, Cathy Fernandes, Adina T. Michael-Titus, Adrian J. Hobbs

**Affiliations:** ^1^William Harvey Research Institute, Barts & The London School of Medicine & Dentistry, Queen Mary University of London, London, United Kingdom; ^2^Centre for Neuroscience, Surgery and Trauma, Blizard Institute, Barts and The London School of Medicine and Dentistry, London, United Kingdom; ^3^Basic and Clinical Neuroscience, Institute of Psychiatry, Psychology and Neuroscience, King’s College London, London, United Kingdom; ^4^Social, Genetic & Developmental Psychiatry Centre, Institute of Psychiatry, Psychology & Neuroscience, King’s College London, London, United Kingdom; ^5^MRC Centre for Neurodevelopmental Disorders, Institute of Psychiatry, Psychology & Neuroscience, King’s College London, London, United Kingdom

**Keywords:** C-type natriuretic peptide, natriuretic peptide receptor C, blood brain barrier, ZO-1, seizure, hyperactivity, anxiety, coordination

## Abstract

C-type natriuretic peptide (CNP) is highly expressed in the central nervous system (CNS) and key to neuronal development; however, a broader role for CNP in the CNS remains unclear. To address this deficit, we investigated behavioral, sensory and motor abnormalities and blood-brain barrier (BBB) integrity in a unique mouse model with inducible, global deletion of CNP (gbCNP^–/–^). gbCNP^–/–^ mice and wild-type littermates at 12 (young adult) and 65 (aged) weeks of age were investigated for changes in gait and motor coordination (CatWalk*™* and rotarod tests), anxiety-like behavior (open field and elevated zero maze tests), and motor and sensory function (modified neurological severity score [mNSS] and primary SHIRPA screen). Vascular permeability was assessed *in vivo* (Miles assay) with complementary *in vitro* studies conducted in primary murine brain endothelial cells. Young adult gbCNP^–/–^ mice had normal gait but reduced motor coordination, increased locomotor activity in the open field and elevated zero maze, and had a higher mNSS score. Aged gbCNP^–/–^ animals developed recurrent spontaneous seizures and had impaired gait and wide-ranging motor and sensory dysfunction. Young adult and aged gbCNP^–/–^ mice exhibited increased BBB permeability, which was partially restored *in vitro* by CNP administration. Cultured brain endothelial cells from gbCNP^–/–^ mice had an abnormal ZO-1 protein distribution. These data suggest that lack of CNP in the CNS impairs tight junction protein arrangement and increases BBB permeability, which is associated with changes in locomotor activity, motor coordination and late-onset seizures.

## Introduction

The blood-brain barrier (BBB) is a highly selective, semipermeable interface that limits the movement of molecules and inflammatory cells from the peripheral circulation into the central nervous system (CNS) (Obermeier et al., 2013). This important, specialized role is enabled by adherens junctions (which include proteins such as VE-cadherin) and tight junctions (which include proteins such as occludin, claudin-5 and ZO-1) that maintain a tight barrier between endothelial cells in the brain microvessels (Kadry et al., 2020). The integrity of BBB declines with aging (Banks et al., 2021). Moreover, various neurological disorders, such as Huntington’s disease, Parkinson’s disease and Alzheimer’s disease, epilepsy, hypoxic and ischemic injury, brain tumors, multiple sclerosis and also chemical poisoning are associated with tight junction protein dysregulation, leading to BBB breakdown. This causes a disruption in the exchange of solutes between the blood and the CNS fluid (Kadry et al., 2020). BBB breakdown can lead to changes in behavior, cognition, and/or induce seizures, as a result of neuronal dysfunction, neuroinflammation, and neurodegeneration (Weissberg et al., 2011; Takeda et al., 2014; Yang et al., 2016). Therefore, a better understanding of the factors that preserve BBB integrity should aid in the development of novel therapeutics for such disorders.

C-type natriuretic peptide (CNP) plays key roles in the regulation of cardiac function and in fine-tuning of the local blood flow, including in cerebral arterioles (Mori et al., 1997; Moyes and Hobbs, 2019). CNP was first identified in porcine CNS (Sudoh et al., 1990) and, in addition to vascular endothelial cells, CNP is most highly expressed in the CNS in mammals, including humans (Kaneko et al., 1993). There is a particularly strong expression of CNP in the hippocampus (Langub et al., 1995), medulla-pons and cerebellum (Zumft, 1997), where the cognate receptors for CNP, i.e., natriuretic peptide receptor (NPR)-B and NPR-C, are also highly expressed (Minamino et al., 1993; Totsune et al., 1994; Mahinrad et al., 2018). The role(s) of CNP in the CNS have been established in certain contexts. For example, CNP regulates sensory neuron branching within the dorsal root ganglia, cranial sensory ganglia and mesencephalic trigeminal neurons during development, via NPR-B/cGMP signaling (Schmidt et al., 2009; Decker et al., 2010; Dumoulin et al., 2018; Schmidt et al., 2018; Tröster et al., 2018; Wolter et al., 2018; Schmidt and Fritzsch, 2019; Dumoulin et al., 2021). In addition, some studies have suggested that CNP acts as a neuromodulator (Charles et al., 1992; Bohara et al., 2014; Ceylan et al., 2018; Li et al., 2019), neuroprotector (Ma and Zhang, 2018; Giovannini et al., 2021), and modulates neuronal plasticity (Barmashenko et al., 2014; Rapley et al., 2018). CNP also exerts central effects to regulate blood pressure (Yamamoto et al., 1997). Interestingly, some of these CNS activities, such as in anxiety (Bíró et al., 1996; Montkowski et al., 1998), vascular permeability (Vigne and Frelin, 1992; Pedram et al., 2002) and neural plasticity (Barmashenko et al., 2014; Rapley et al., 2018), have shown contrasting effects, depending on the targeted receptor or the pharmacological dose used.

To advance understanding of the physiological roles of CNP in the CNS and the integrity of the BBB, we have characterized the behavior and blood-brain barrier permeability of young adult and aged mice using a novel transgenic mouse model with inducible, global CNP deletion.

## Materials and methods

### Animals and husbandry

Global CNP knockout mice (gbCNP^–/–^) were generated by crossing *Nppc* LoxP-flanked (CNP*^flox/flox^*) with a tamoxifen-inducible universal Cre deleter line (B6.Cg-Tg[UBC-cre/ERT2]1Ejb/1J; The Jackson Laboratory, Bar Harbor, USA), as previously described (Perez-Ternero et al., 2022). Mice were weaned at 28 days of age and CNP gene excision was induced by tamoxifen injections (40 mg/kg/day, i.p.) on 5 consecutive days at 5 weeks of age. Wild-type (WT) Cre positive animals receiving tamoxifen injections were used as control. Studies were conducted on 12- (young adult) and 65- (aged) week-old mice of both sexes. Mice were group-housed by sex in individually ventilated cages, equipped with cardboard bio-tunnels, sawdust and nesting material (concertina-folded paper strips), kept at 21°C and 45% humidity with a 12-h light-dark cycle (lights on from 7 a.m. to 7 p.m.) and free access to food (5,058, LabDiet, St. Louis, USA) and water. For all experiments, the investigator was blinded to the genotype whenever possible. Some animals were used for biochemical studies following behavioral tests.

### Behavioral tests

The age of the animals at which behavioral tests were performed is provided in Table 1.

**TABLE 1:** Age of animals studied in each behavioral test.

	Young adult (12 weeks old)	Aged (65 weeks old)
*CNP expression characterization* *(Figure 1)*	X	
*Rotarod* *(Figure 2)*	X	
*mNSS* *(Figure 2)*	X	
*CatWalk™* *(Figure 2)*	X	
*Open Field* *(Figure 3)*	X	
*Zero maze* *(Figure 3)*	X	
*Seizures* *(Figure 4)*	X	X
*Limb clasping* *(Figure 4)*		X
*SHIRPA test* *(Figure 5)*		X
*Miles assay* *(Figure 6)*	X	X

#### Catwalk*™* gait analysis

Gait and locomotor coordination were assessed in 12-week-old animals using the CatWalk*™* gait analysis system (Noldus Information Technology, Leesburg, VA, USA). Footprints of mice walking on a green LED illuminated glass plate were recorded by a high-speed color camera positioned underneath, which allows measuring the time, dimensions, and position (stand and swing times, stride length, print size, etc.) of every footprint along with parameters related to gait coordination (cycle patterns, regularity index, phase dispersion, etc.). Mice were trained on the apparatus over a minimum of 3 trials on the day before data acquisition. On testing day, runs where the animal had turned or stopped were excluded. Runs exceeding 60% speed variation were defined as non-compliant and were discarded. A minimum of 2 runs were analyzed per animal using the CatWalk^XT^ 10.5 software.

#### Rotarod

Gross motor abilities and coordination were assessed using a rotarod device (Med Associates Inc; Georgia, Vermont, USA). Young adult mice were trained and tested over 4 consecutive days, each day consisting of 3 trials. During the first two trials, the mice were allowed to familiarize themselves with the apparatus by letting them walk for 60 s (trial 1) or 5 min (trial 2) at the lowest speed of 4 rpm. For trial 3, the mice would start by walking at 4 rpm for 30 s, after which the speed would be increased progressively to 40 rpm over 5 min. On the 4th day, a fixed speed version of the test was performed after testing the mice in accelerating mode. For fixed speed rotarod, the mice were placed on the rod for 60 s for each trial and were tested twice at each speed, starting with the lowest speed before moving to the next one, in the following order: 4, 16, 20, 24, 28, 32, and 40 rpm. In all instances, the time at which the mice fell was recorded; if a mouse stayed on the rod throughout, the maximum time of the trial duration was recorded as the trial performance. Inter-trial intervals were 5 to 10 min. The mean time at which each mouse fell in accelerating mode was plotted across testing days for accelerating rotarod; for fixed speed rotarod, the average of the 2 trials was plotted at each walking speed.

#### Modified neurological severity score

Gross motor function, alertness, balance, and other indexes of neurological health status were evaluated in 12-week-old mice for 5 consecutive days using the modified Neurological Severity Score (mNSS) (Tsenter et al., 2008). The mNSS assigns a score for every task that the mice fail to complete, implying that the higher the score the higher the neurological impairment.

#### Open field test

The open field test is used to assess anxiety-like behavior, locomotor activity and exploratory behavior in rodents and other mammals. It is based on an approach/avoidance conflict between an animal’s tendency to explore a novel area and its avoidance of the aversive nature of the environment (openness), with avoidance of the novel, potentially threatening center of the arena being interpreted as a measure of anxiety. The test apparatus consisted of a square chamber (50 cm × 50 cm × 50 cm, l x w x h) laid in a quiet environment with black opaque walls and a white floor under indirect illumination, resulting in light intensity of 15 Lux in the center of the chamber and 13 Lux in each corner. 12-week old mice were tested on the open field. Each mouse was gently placed facing always the same wall of the arena at the start of each trial and was then allowed to explore freely for 10 min. Three regions were arbitrarily defined; the center and inner regions were delineated as the 10 × 10 cm and 30 × 30 cm areas in the center of the arena, respectively, and the periphery of the test was defined as the remaining area closest to the walls. The locomotor reactivity to the test was recorded and analyzed using the ANY-maze software (Stoelting Europe, Dublin, Ireland). Total distance traveled, average speed, number of zone entries were computed automatically by the software, while grooming (duration and bout frequency), supported (against an arena wall) and unsupported (in the open areas of the arena) rearing frequencies, were recorded by the investigator.

#### Elevated zero maze

Anxiety-like behavior was further evaluated by assessing exploratory behavior in another approach/avoidance conflict-based task, the elevated zero maze (Shepherd et al., 1994). The maze consisted of a 105 cm in diameter and 10-cm wide annular platform elevated 65 cm from the ground and was equipped with two opposite enclosed (1 Lux) and two opposite open (3 Lux) quadrants. Such design allows uninterrupted exploration of the four quadrants, with the mice being able to alternate visits between enclosed and open quadrants, and offers four zones of conflict, each being the interface of a safe, enclosed quadrant with a potentially threatening elevated open quadrant. To start the trial, the mice were gently placed in the center of always the same enclosed quadrant, after which they were allowed to freely explore the maze for 5 min. Avoidance of the open quadrants, along with the display of ethologically-relevant behaviors -stretched attend postures (SAPs) from the closed to open quadrants, which are positively correlated with anxiety, and head dips (HDIPs) over the edge of the open arms, which are negatively correlated with anxiety, were used as indicators of the presence of an anxiety-like profile. Locomotor reactivity to the test was recorded and analyzed using the ANY-maze software (Stoelting Europe, Dublin, Ireland) and HDIPs from the closed or open areas and SAPs were recorded by the investigator.

#### Seizure and limb clasping

Handling-induced seizures were monitored weekly and scored according to the Racine scale (Racine, 1972): 0 – No seizure; 1 – Immobility, twitching; 2 – Head nodding; 3- Clonus of one forelimb; 4 – Bilateral forelimb clonus with rearing; 5 – Falling on a side, tonic-clonic seizures. The age at which the first handling-induced seizure occurred was noted as the age of seizure onset, and the incidence of seizures (as percentage) was computed as the number of events induced by handling over the total number of times that the mice were handled. Every mouse was assigned the highest Racine severity score that they had reached in their lifetime.

Adult mice suspended by their tail extend their four limbs in anticipation of landing. Limb clasping, often seen in a number of neurodegenerative and motor dysfunction models, was assessed in young adult mice as part of the mNSS test (see above) and in aged mice, as described previously (Miedel et al., 2017).

#### Primary SHIRPA screen

Gross phenotype assessment of motor and sensory function of aged mice was performed using elements of the primary screen of the SmithKline, Harwell, Imperial College, Royal Hospital, Phenotype Assessment (SHIRPA) test (Rogers et al., 1997; Lalonde et al., 2021). This is a set of functional assessments that are designed to identify and systematically quantify abnormalities in murine muscle tone, lower motor neuron and spinocerebellar function, sensory and autonomic ability and motor coordination/capabilities. The test was performed once in 65-week-old mice, in a quiet room, and scores were assigned to every assessment based on numeric rating scales.

### Miles assay

Vascular extravasation was evaluated by measuring the extravasation of Evans blue dye from the circulatory system into the interstitial tissue. Evans blue (120 μg/kg; Sigma, UK) was administered intravenously and animals were sacrificed 45 min after the injection. To avoid any interference of dye remaining in the circulatory system, mice were anesthetized using isoflurane and perfused with saline until the outflow fluid was colorless. Organs were then dissected, weighed, and incubated in formamide at 55°C overnight. After centrifugation at 18,620 g for 2 min, the absorbance of the supernatant was measured at 620 nm, and the relative Evans blue content in the tissue was expressed as ng Evans blue per mg of tissue.

### Murine brain endothelial cell culture and extravasation assay

Murine brain endothelial cells (MBEC) were isolated as previously described (Assmann et al., 2017) and maintained in DMEM-F12 (Gibco, ThermoFisher Scientific, UK) supplemented with 10% fetal bovine serum (Sigma, UK), and 1% antibiotic/antimycotic (Gibco, ThermoFisher Scientific, UK), 200 mM L-glutamine (Gibco, ThermoFisher Scientific, UK), 5000 U/ml heparin (Leo Pharma, Denmark) and 30 μg/ml endothelial cell growth supplement (Sigma, UK). The integrity of the endothelial cell monolayer was tested by measuring the diffusive permeability of fluorescein isothiocyanate (FITC)-albumin. Endothelial cells were grown in 6.5 mm in diameter, collagen-coated transwells (Corning, New York, USA) and allowed to form a monolayer. FITC-albumin (1 mg/ml) was then added to the top chamber and after 10 min a sample was taken from the lower chamber to measure basal extravasation. Thrombin (5 IU/ml; Sigma, UK) was then added alone or in the presence of CNP or cANF^4–23^ (both 100 nM), and samples from the lower chamber were taken every 15 min for 1 h. Extravasation was expressed as fold change over the basal extravasation (i.e., in the absence of thrombin).

### mRNA quantification

Mice were anesthetized with isoflurane and sacrificed by exsanguination. Whole brains were homogenized in liquid nitrogen and total RNA was extracted from 10 mg of tissue using RNeasy Lipid Tissue Mini Kit (Qiagen, UK), as instructed by the manufacturer. cDNA was transcribed from 1 μg of total RNA using a high-capacity cDNA Reverse Transcription Kit (Applied Biosystems, Waltham, Massachusetts, USA), and the resulting cDNA was subjected to PCR using PowerUp™ SYBR™ Green (Applied Biosystems, Life technologies Ltd., UK) and the gene-specific primers listed in Table 2 on a Bio-Rad CFX96 Connect Real-Time PCR detection system (Bio-Rad, California, USA). The relative expression level was expressed as fold change vs WT, using the 2^–ΔΔ^ ^Ct^ method and *Rpl-19* expression as an internal control.

**TABLE 2:** Mouse primer sequences with corresponding database reference.

Gene/primer	Sequence (5′→3′)	References
***Mouse CNP (Nppc) Forward***	CCAACGCGCGCAAATACAAA	NM_010933.5
***Mouse CNP (Nppc) Reverse***	GCACAGAGCAGTTCCCAATC	
***Mouse Claudin-5 (Cldn5) Forward***	CCCAGTTAAGGCACGGGTAG	NM_013805.4
***Mouse Claudin-5 (Cldn5) Reverse***	GGCACCGTCGGATCATAGAA	
***Mouse NPR-B (Npr2) Forward***	AACGGGCGCATTGTGTATATCT	NM_173788.4 NM_001355466.1
***Mouse NPR-B (Npr2) Reverse***	TCAGGATTTGGGGGTTCTCG	
***Mouse NPR-C (Npr3) Forward***	CTTGGATGTAGCGCACTATGTC	NM_001039181.1 NM_008728.2
***Mouse NPR-C (Npr3) Reverse***	CACAAGGACACGGAATACTC	
***Mouse Occludin (Ocln) Forward***	GGTTGATCCCCAGGAGGCTA	NM_001360538.1 NM_001360536.1 NM_001360537.1 NM_008756.2
***Mouse Occludin (Ocln) Reverse***	GCTTGCCATTCACTTTGCCA	
***Mouse Rpl19 (Rpl19) Forward***	TTGGCGATTTCATTGGTCTCA	NM_009078.2 NM_001159483.1
***Mouse Rpl19 (Rpl19) Reverse***	GCTTGCCTCTAGTGTCCTCC	
***Mouse VE-Cadherin (Cdh5) Forward***	GCTCACGGACAAGATCAGCTC	NM_009868.4
***Mouse VE-Cadherin (Cdh5) Reverse***	ACTTAGCATTCTGGCGGTTCA	
***Mouse ZO-1 (Tjp1) Forward***	GCGCGGAGAGAGACAAGATGT	NM_001163574.1 NM_009386.2
***Mouse ZO-1 (Tjp1) Reverse***	CAACTCGGTCATTTTCCTGTAGC	

### Immunohistochemistry

Mice were anesthetized with isoflurane and sacrificed by exsanguination. Then, the animals were retrogradely perfused with saline and 4% paraformaldehyde (PFA). Immunohistochemical analysis of murine brains and brain microvascular endothelial cells was performed on PFA-perfused brains embedded in OCT (10 μM-thick sections) and in PFA-fixed cell cultures, following standard protocols (Moyes et al., 2014; Bubb et al., 2019). Primary antibodies against CNP (1:1000 for cells, 1:100 for tissue; Cat# H-012-03, Phoenix Pharmaceuticals, Karlsruhe, Germany), CD-31 (1:1000; Cat# 14-0311-81 eBioscience, San Diego, CA, USA), alpha smooth muscle actin (α-SMA) (1:1000; Cat# ab5694, Abcam, Cambridge, UK), ZO-1 (1:100; Cat# 10017242, Fisher Scientific Uk Ltd., Leicestershire, UK), occludin (1:100; Cat# ab216327, Abcam, Cambridge, UK), VE-cadherin (1:100; Cat# ab205336, Abcam, Cambridge, UK) and claudin-5 (1:100; Cat# 10681224, Fisher Scientific UK Ltd., Leicestershire, UK) were incubated with tissue or cells overnight and incubation with the secondary antibodies (biotin-conjugated Donkey anti-Rabbit, Cat# 31821, ThermoFisher Scientific, UK, 1:500 for tissue; Alexa Fluor 488 (Cat# A21208 and A11008) and 546 (Cat# A11081), ThermoFisher Scientific, UK, 1:1000 for cells) was performed at room temperature the following day. Whole brain sections from DAB-stained samples were imaged using a × 40 objective in a NanoZoomer S210 Slide Scanner (Hamamatsu, Japan) and immunofluorescence samples using a Zeiss LSM 880 with FAST Airyscan (Zeiss, Germany). Densitometric analysis of DAB images was performed with the image analysis Fiji software (Image J; NIH, USA).

### Experimental design and statistical analysis

The investigator was blinded to genotype whenever possible. Although these studies used both male and female animals they were not sufficiently powered to detect potential sex differences; however, no overt sex-dependent changes were noted. All statistical analyses were performed with GraphPad Prism (version 9; GraphPad Software, California, USA). For simple comparison of WT and knockout mice, normal distribution of the data sets was assessed by a Shapiro-Wilk test and, depending on the outcome of the test, a two-tailed, unpaired Student’s *t*-test or a Mann–Whitney *U* test was conducted. When comparing three or more groups of data one-way or two-way ANOVA followed by a Šídák multiple comparisons test was used with adjustment for multiplicity. All data were expressed as mean ± SEM. Statistical significance was set at *P* < 0.05 and the *P* values presented in each Figure indicate all comparisons undertaken.

## Results

### The effect of CNP deletion on body weight, muscle weight and the expression of CNP and its receptors

**FIGURE 1:**
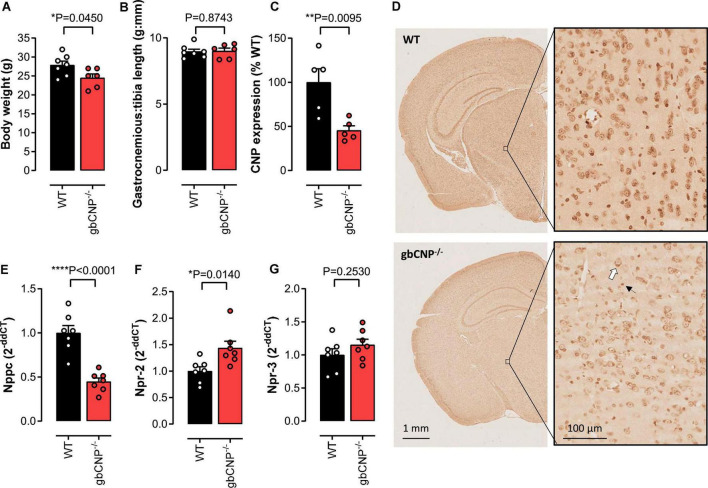
The effect of CNP deletion on body weight, muscle weight and the expression of CNP and its receptors. Body weight **(A)** and gastrocnemius weight normalized to tibia length **(B)** in wild-type (WT) and global CNP knockout (gbCNP^– /–^) mice at 12 weeks of age. Densitometric quantification of CNP protein expression using diaminobenzidine (DAB) immunohistochemistry in the brain of WT and gbCNP^– /–^ mice **(C)** and representative images **(D**, white arrow indicates neuron and black arrow microglia). CNP (*Nppc*, **E**), NPR-B (*Npr2*, **F)** and NPR-C (*Npr3*, **G)** mRNA expression in brain homogenates. *n* = 5-7. Statistical analysis by two-tailed Student’s *t*-test. Each statistical comparison undertaken has an assigned *P* value (adjusted for multiplicity).

Global CNP^–/–^ mice were characterized by reduced body weight, as we have previously reported (Perez-Ternero et al., 2022; Figure 1A); however, gastrocnemius weight, an indicator of muscle mass, remained unchanged (Figure 1B). Next, we evaluated the expression of CNP in cross sectional brain sections from WT and gbCNP^–/–^ mice. The peptide was mainly expressed in the soma of neurons and neuroglia (distinguishable by size) and overall CNP expression was reduced by 54.5 ± 5.2% in the brain of transgenic mice (Figures 1C,D), which is commensurate with the deletion achieved in this conditional knockout strain in other tissues, as we have previously reported (Perez-Ternero et al., 2022). This level of downregulation was confirmed by qPCR in whole brain homogenates (Figure 1E). Next, we evaluated the expression of the CNP cognate receptors NPR-B and NPR-C, by quantifying the expression levels of their mRNAs, Npr-2 and Npr-3, respectively. Interestingly, while the expression of NPR-B was significantly upregulated in gbCNP^–/–^ mice (Figure 1F), NPR-C expression remained unchanged (Figure 1G).

### Young adult gbCNP^–/–^ mice showed normal gait and motor coordination when walking overground but not when their balance was challenged

**FIGURE 2:**
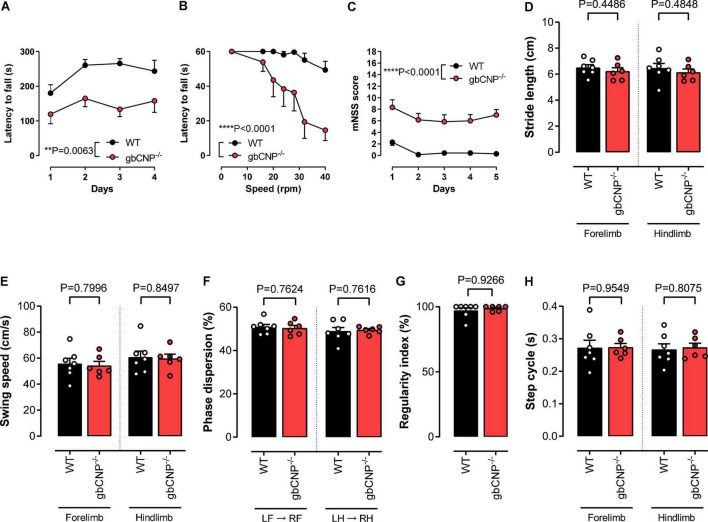
Young adult gbCNP^– /–^ mice showed normal gait and motor coordination when walking overground but not when their balance was challenged. Rotarod latency to fall when performing in accelerating **(A)** and fixed speed **(B)** modes. Modified neurological severity score (mNSS, **C)**. CatWalk™ analysis of gait and coordination in wild-type (WT) and global CNP knockout (gbCNP^– /–^) mice. Stride length **(D)**, Swing speed **(E)**, Phase dispersion **(F)**, Regularity index **(G)** and Step cycle **(H)**. *n* = 6-7. Statistical analysis by two-tailed Student’s *t*-test, Mann–Whitney *U* test or two-way analysis of variance with Šídák *post hoc* test. Each statistical comparison undertaken has an assigned *P* value (adjusted for multiplicity).

In order to evaluate locomotor ability in gbCNP^–/–^ mice, we first assessed the performance of the mice on the accelerating and fixed speed versions of the rotarod. In accelerating mode, the majority of WT mice were able to complete the task, reaching the speed of 40 rpm within 300 s (Figure 2A). However, gbCNP^–/–^ mice had a significantly shorter latency to fall, despite a slight improvement in the performance compared to the first day of exposure to the apparatus (Figure 2A). On the fixed speed version of the task, both genotypes were able to complete the task at the lowest speed (4 rpm). However, as the speed was incrementally increased, the latency to fall decreased correspondingly in gbCNP^–/–^ mice. In contrast, WT mice were able to complete the test at all but the highest speeds (32 and 40 rpm; Figure 2B). In the light of the altered performance of the knockout mice on the rotarod, which could potentially have various origins, sensorimotor performance was further assessed using the modified neurological severity score (mNSS) for mice. Indeed, gbCNP^–/–^ mice showed significantly impaired performance in some of the mNSS tasks assessing motor abilities, such as walking on a beam or balancing on a round or triangular stick (Figure 2C and Supplementary Figure 1). Importantly, gbCNP^–/–^ mice also showed a marked limb clasping when suspended by the tail, characterized by a retraction of both limbs toward the body, as opposed to the normal extension reflex observed in WT animals (Supplementary Figure 1). The detailed characteristics of their gait were further assessed in the CatWalk*™* apparatus. The main gait parameters and those linked to gait coordination in gbCNP^–/–^ mice did not differ to these of their WT littermates (Figures 2D–H and Supplementary Figure 2). These data show that gbCNP^–/–^ mice have impaired coordination and abnormal motor reflexes that are exacerbated in tasks with increased complexity.

### Young adult gbCNP^–/–^ mice were more active than their wild-type littermates in the open field and elevated zero maze

**FIGURE 3:**
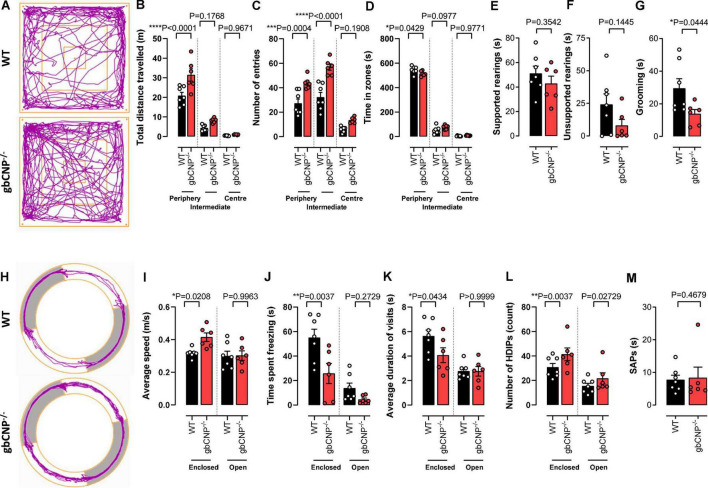
Young adult gbCNP^– /–^ mice were more active than their wild-type littermates in the open field and elevated zero maze. Open field analyses in wild-type (WT) and global CNP knockout (gbCNP^– /–^) mice. Representative traces of movement of the mice in the apparatus **(A)**, total distance traveled **(B)**, number of entries in each zone of the open field **(C)**, time spent in each zone of the open field **(D)**, time spent on supported **(E)** and unsupported **(F)** rearings, and time spent grooming **(G)**. Elevated zero maze analyses in WT and gbCNP^/^ mice: Representative traces of movement of the mice in the apparatus **(H)**, average speed **(I)**, time spent freezing **(J)**, average duration of the visits **(K)**, head dips (HDIPs) **(L)** and stretching attend postures (SAPs, **M)**. *n* = 6-7. Statistical analysis by two-tailed Student’s *t*-test, Mann–Whitney *U* test or two-way analysis of variance with Šídák *post hoc* test. Each statistical comparison undertaken has an assigned *P* value (adjusted for multiplicity).

To explore anxiety-like behavior, 12-week-old mice were tested in the open field and elevated zero maze tests. gbCNP^–/–^ mice were more active, traveled longer distances and had a lower number of immobile episodes compared to WT (Figures 3A,B and Table 3).

**TABLE 3:** Open field behavior in wild-type and global CNP knockout mice.

	Periphery		
	WT	gbCNP^–/–^	P value
***Total distance travelled (m)***	20.81 ± 1.75	31.33 ± 3.08	**** < 0.0001
***Total time mobile (s)***	351.00 ± 21.80	453.60 ± 18.85	**** < 0.0001
***Number of immobile episodes (count)***	26.86 ± 1.87	11.67 ± 1.48	**** < 0.0001
***Average speed (m/s)***	0.038 ± 0.003	0.061 ± 0.005	0.5363
***Number of entries (count)***	27.14 ± 3.61	44 ± 2.03	***0.0004
***Time in zones (s)***	544.50 ± 11.48	514.30 ± 8.60	*0.0429
***Absolute turn angle (degrees)***	43197 ± 3139	57755 ± 4354	***0.0001

	**Intermediate**		
	
	**WT**	**gbCNP^–/–^**	**P value**

***Total distance travelled (m)***	4.31 ± 0.54	8.204 ± 0.50	0.1768
***Total time mobile (s)***	34.77 ± 5.12	70.57 ± 4.98	0.1387
***Number of immobile episodes (count)***	4.14 ± 1.44	3.00 ± 1.55	0.906
***Average speed (m/s)***	0.10 ± 0.02	0.11 ± 0.01	0.9078
***Number of entries (count)***	32.14 ± 4.00	56.83 ± 2.64	**** < 0.0001
***Time in zones (s)***	51.19 ± 11.05	77.13 ± 7.32	0.0977
***Latency to first entry (s)***	23.63 ± 5.35	42.10 ± 17.24	0.8668
***Absolute turn angle (degrees)***	4148 ± 717	7182 ± 595	0.5547

	**Centre**		
	
	**WT**	**gbCNP^–/–^**	**P value**

***Total distance travelled (m)***	0.14 ± 0.14	0.99 ± 0.07	0.9671
***Total time mobile (s)***	3.71 ± 0.81	8.00 ± 1.45	0.9928
***Number of immobile episodes (count)***	0.14 ± 0.14	0.17 ± 0.17	> 0.9999
***Average speed (m/s)***	0.11 ± 0.01	0.13 ± 0.01	0.8927
***Number of entries (count)***	6.00 ± 0.87	13.33 ± 0.99	0.1908
***Time in zones (s)***	4.26 ± 1.14	8.55 ± 1.51	0.9771
***Latency to first entry (s)***	79.50 ± 16.41	61.10 ± 13.58	> 0.9999
***Absolute turn angle (degrees)***	416.00 ± 105.20	986.20 ± 99.59	0.8546

	**Mean for the full open field**		
	
	**WT**	**gbCNP^–/–^**	**P value**

***Latency to first unsupported rearing (s)***	204.10 ± 34.86	280.10 ± 74.69	0.3533
***Number of unsupported rearings (count)***	25.43 ± 8.56	10.50 ± 6.33	0.2774
***Time spent doing unsupported rearings (s)***	24.09 ± 8.12	7.95 ± 4.71	0.1445
***Latency to first supported rearing (s)***	16.84 ± 6.40	29.00 ± 4.96	0.0513
***Number of supported rearings (count)***	53.86 ± 6.23	67.00 ± 8.47	0.2287
***Time spent doing supported rearings (s)***	51.06 ± 6.00	42.73 ± 6.12	0.3542
***Latency to first grooming episode (s)***	133.10 ± 21.81	124.50 ± 31.91	0.823
***Number of grooming bouts (count)***	15.14 ± 4.11	12.67 ± 3.05	0.7045
***Time spent doing grooming (s)***	29.41 ± 5.90	13.83 ± 2.67	*0.0444
***Time freezing (s)***	11.13 ± 3.88	7.83 ± 2.27	0.486

Data are represented as the mean SEM. *n* = 6-7. two-way analysis of variance with Šídák post hoc test. *P < 0.05, ***P < 0.001 and ****P < 0.0001 significantly different from WT.

This hyperactive behavior was also apparent in the higher number of entries to the intermediate and peripheral areas of the open field (Figure 3C). Moreover, gbCNP^–/–^ mice spent less time in the periphery, considered a less anxiogenic area (Figure 3D and Table 3). Supported and unsupported rearings are, in addition of a index of locomotor activity, a manifestation of the natural exploratory behavior in mice, and are responsive to acute stress (Sturman et al., 2018). Similarly, changes in self-grooming patterns may indicate aversiveness (Fernández-Teruel and Estanislau, 2016) and neurological disease (Kalueff et al., 2016). Supported and unsupported rearing were similar in WT and gbCNP^–/–^ mice (Figures 3E,F and Table 3). However, CNP null mice, despite having a similar frequency of grooming bouts (Table 3), spent less time grooming compared to WT counterparts (Figure 3G). gbCNP^–/–^ mice were also more active in the zero maze task, travelled at a higher speed and spent less time freezing (Figures 3H–J and Table 4). There were no differences in the number of entries into, or time spent in the open arms or distance traveled in this zone between WT and gbCNP^–/–^ mice (Table 4). However, gbCNP^–/–^ mice visits to the enclosed arm were shorter (Figure 3K). Head dips (HDIPs) from the open and close arms and stretched attend postures (SAP) are exploratory behaviors that serve as indices of anxiety (Braun et al., 2011). WT and gbCNP^–/–^ mice exhibited a similar number of HDIPs and SAPs (Figures 3L,M and Table 4). Overall, the data from open field and zero maze tasks indicate that gbCNP^–/–^ mice display increased locomotor activity in the open field and elevated zero maze but unaltered anxiogenic profile.

**TABLE 4:** Elevated zero maze behavior in wild-type and global CNP knockout mice.

	Enclosed arms		
	WT	gbCNP^–/–^	P value
***Total distance travelled (m)***	63.25 ± 3.27	72.51 ± 4.58	0.2915
***Average speed (m/s)***	0.32 ± 0.01	0.41 ± 0.03	*0.0208
***Time in zones (s)***	199.50 ± 7.30	177.70 ± 14.35	0.3072
***Average duration of visits (s)***	5.62 ± 0.50	4.07 ± 0.61	*0.0434
***Absolute turn angle (degree)***	15624 ± 962	16748 ± 2362	0.794
***Total time mobile (s)***	129.00 ± 8.50	147.00 ± 11.51	0.2626
***Number of immobile episodes***	20.29 ± 2.99	11.00 ± 1.53	0.0592
***Number of freezing episodes***	23.29 ± 3.07	12.33 ± 3.08	*0.0220
***Time spent freezing (s)***	54.93 ± 6.99	25.73 ± 8.22	**0.0037
***Distance traveled before 1st HDIPs from closed arms (m)***	1.68 ± 0.51	6.21 ± 1.97	**0.0047
***Latency to 1st HDIPs from closed arms (s)***	5.94 ± 1.32	15.15 ± 4.75	*0.0221
***Number of HDIPs***	30.71 ± 3.16	41.33 ± 5.21	0.1203
***Mean frequency of doing HDIPs (s)***	10.24 ± 1.06	13.78 ± 1.73	0.5244
***Time spent doing HDIPs (s)***	42.60 ± 7.76	35.55 ± 5.02	0.5977
***Mean duration of HDIPs (s)***	1.40 ± 0.27	0.88 ± 0.12	0.114
***Latency to 1st SAPs (s)***	10.41 ± 2.20	13.63 ± 2.39	0.3418
***Distance traveled before 1st SAPs (m)***	4.01 ± 1.27	5.45 ± 1.06	0.4151
***Number of SAPs***	15.57 ± 3.28	16.83 ± 4.95	> 0.9999
***Frequency of doing SAPs***	0.05 ± 0.01	0.06 ± 0.02	> 0.9999
***Time spent doing SAPs (s)***	7.71 ± 1.42	8.35 ± 3.27	0.4679
***Mean duration of SAPs (s)***	0.50 ± 0.02	0.45 ± 0.03	0.1579

	**Open arms**		
	
	**WT**	**gbCNP^–/–^**	**P value**

***Total distance traveled (m)***	30.14 ± 4.04	37.83 ± 6.04	0.4196
***Average speed (m/s)***	0.30 ± 0.03	0.30 ± 0.03	0.9963
***Time in zones (s)***	99.67 ± 6.76	122.10 ± 14.41	0.2891
***Average duration of visits (s)***	2.76 ± 0.24	2.75 ± 0.39	> 0.9999
***Absolute turn angle (degree)***	7119 ± 267	11044 ± 991	0.0852
***Total time mobile (s)***	74.89 ± 2.84	100.20 ± 9.32	0.0857
***Number of immobile episodes***	11.71 ± 2.84	9.83 ± 2.46	> 0.9999
***Number of freezing episodes***	12.00 ± 2.92	5.67 ± 1.52	0.123
***Time spent freezing (s)***	13.73 ± 4.12	4.45 ± 1.27	0.2729
***Latency to 1st entry to open arm***	2.84 ± 1.67	3.43 ± 2.18	0.7273
***Distance traveled before 1st entry to open arm (m)***	0.59 ± 0.37	1.52 ± 0.98	0.3384
***Number of entries in open arms***	36.86 ± 2.95	46.50 ± 5.32	0.1929
***Number of HDIPs***	15.43 ± 2.24	21.67 ± 4.57	0.4532
***Mean frequency of doing HDIPs (s)***	5.14 ± 0.74	7.22 ± 1.51	0.7158
***Time spent doing HDIPs (s)***	17.29 ± 3.46	16.88 ± 3.44	0.9584
***Mean duration of HDIPs (s)***	1.14 ± 0.15	0.82 ± 0.10	0.2214
***Latency to 1st HDIPs from open arms (s)***	37.53 ± 14.77	45.07 ± 11.30	0.701
***Distance traveled before 1st HDIPs from open arms (m)***	10.58 ± 4.21	17.18 ± 4.40	0.3033

Data are represented as the mean SEM. *n* = 6-7. two-way analysis of variance with Šídák post hoc test. *P < 0.05 and **P < 0.01 significantly different from WT.

### Aged gbCNP^–/–^ mice exhibited seizures

**FIGURE 4:**
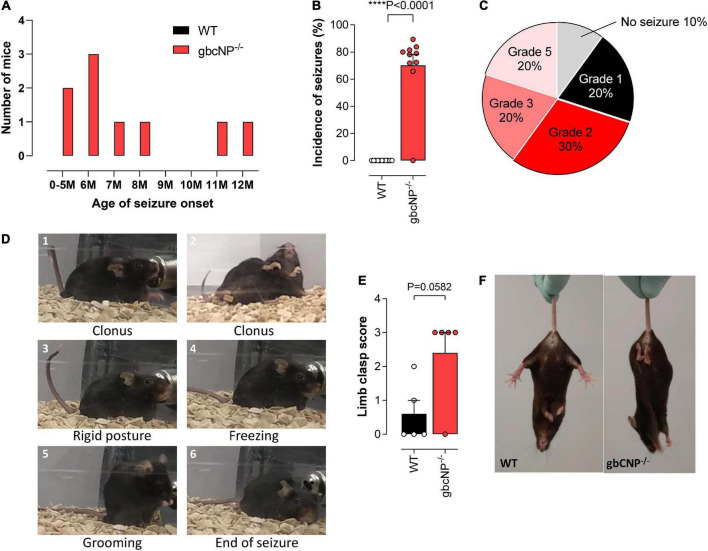
Aged gbCNP^– /–^ mice exhibited seizures. Age of seizure onset in months (M) in wild-type (WT) and global CNP knockout (gbCNP^– /–^) mice **(A)**. Incidence of seizures **(B)**. Distribution of the seizure severity according to the Racine scale (0 – No seizure; 1 – Immobility, twitching; 2 – Head nodding; 3- Clonus of one forelimb; 4 – Bilateral forelimb clonus with rearing; 5 – Falling on a side, tonic-clonic seizures) **(C)**. Representative images of a typical seizure in gbCNP^– /–^ mice **(D)**. Limb clasping score **(E)** and representative images **(F)**. *n* = 5-10. Statistical analysis by two-tailed Student’s t-test. Each statistical comparison undertaken has an assigned *P* value (adjusted for multiplicity).

Having identified some abnormal locomotor features in the gbCNP^–/–^ mice, we wondered if these would become more severe with time. This follow-up led to the unexpected discovery that gbCNP^–/–^ mice started to show epileptic-like seizures from 5 months of age, triggered by routine handling during cage changing (Figure 4A). The onset varied from 5 to 12 months of age; 90% of the transgenic mice developed seizure episodes at some point during their lifetime, while none of the WT counterparts showed any signs of seizure. The seizures occurred ∼70% of the times that the animals were handled (Figure 4B). The severity of the seizures varied from freezing and head nodding (Racine scale grade 1) to a tonic-clonic epileptic-like seizure (Racine scale grade 5; Figure 4C)^1^. The epileptic episodes started with freezing, head jerks, rearing and Straub tail, and were followed by tonic convulsions of the forelimbs, or the forelimbs and hindlimbs, often followed by loss of balance, truncal extension, and excessive salivation (Figure 4D). The seizures finished by a freezing episode, followed by grooming and return to normal behavior. Aged gbCNP^–/–^ mice showed marked limb clasping when they were suspended by the tail, characterized by the retraction of both hindlimbs toward the core of the body, which is consistent with some form of neurological impairment (Figures 4E,F).

### Abnormal gait, motor and sensory function in aged gbCNP^–/–^ mice

**FIGURE 5:**
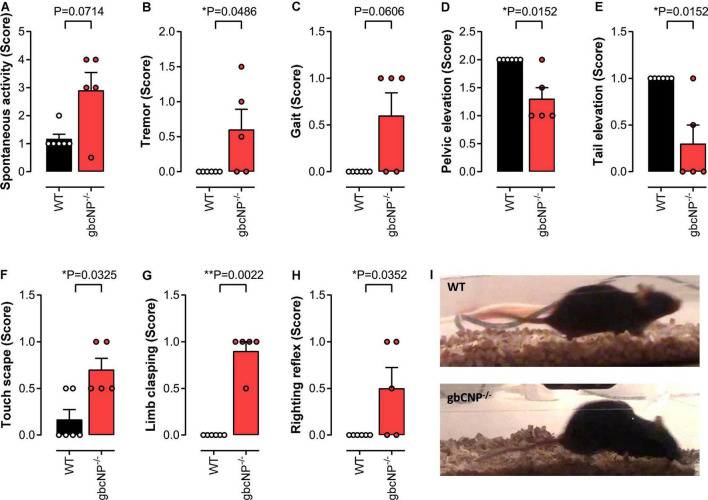
Abnormal gait, motor and sensory function in aged gbCNP^– /–^ mice. Primary screen of SHIRPA in aged (65 weeks) wild type (WT) and global CNP knockout (gbCNP^– /–^) mice. Spontaneous activity **(A)**, tremor **(B)**, gait **(C)**, pelvic elevation **(D)**, tail elevation **(E)**, touch scape **(F)**, limb clasping **(G)**, righting reflex **(H)**, and representative images of WT and gbCNP^– /–^ mice **(I)**. *n* = 5. Statistical analysis by two-tailed Student’s t-test. Each statistical comparison undertaken has an assigned *P* value (adjusted for multiplicity).

The primary screen of SHIRPA can be used in mice for a gross assessment of the motor and sensory phenotype. The increased locomotor activity observed in young adult gbCNP^–/–^ mice was also seen in the aged mice (Figure 5A), which also presented with a marked temporary tremor (Figure 5B) and an abnormal gait (Figure 5C) characterized by reduced pelvic (Figures 5D,I) and tail elevation when walking (Figures 5E,I). Global CNP^–/–^ mice also presented unprovoked episodes of walking backwards or jumping in a forward direction. Transgenic mice also had an increased avoidance to being touched, displaying an increase escape response when the experimenter tried to touch them, compared to their WT counterparts (Figure 5F). Finally, gbCNP^–/–^ mice also showed increased limb clasping (Figure 5G), and impaired righting reflex (Figure 5H) characterized by an uneven posture upon landing (landing on their side or not fully landing on all 4 paws) and presence of freezing episodes. Other general/gross measures scored in SHIRPA (respiration rate, urination and defecation bouts, palpebral closing, startle reflex, rearing, grip strength, fear response, irritability, aggression) were similar between WT and gbCNP^–/–^ mice (Table 5). These data imply that some motor and sensory abnormalities are present in aged gbCNP^–/–^ mice.

**TABLE 5:** SHIRPA score.

	Score	WT	gbCNP^–/–^	*P* value
***Body Position***	0-5	3.50 ± 0.18	3.90 ± 0.24	0.2143
***Spontaneous Activity***	0-4	1.17 ± 0.17	2.90 ± 0.64	*0.0189
***Respiration Rate***	0-3	2.00 ± 0.00	2.00 ± 0.00	N.A.
***Tremor***	0-2	0.00 ± 0.00	0.60 ± 0.29	*0.0486
***Urination***	0-2	0.50 ± 0.50	1.00 ± 0.63	0.5449
***Defecation***	n.	1.67 ± 0.56	1.00 ± 0.55	0.4208
***Transfer Arousal***	0-6	3.17 ± 0.56	3.40 ± 0.40	0.7515
***Locomotor Activity***	n.	16.33 ± 2.30	13.40 ± 2.42	0.4049
***Rearing***	n.	5.50 ± 0.81	3.60 ± 0.51	0.0907
***Palpebral Closing***	0-2	0.00 ± 0.00	0.00 ± 0.00	N.A.
***Piloerection***	0-1	0.00 ± 0.00	0.00 ± 0.00	N.A.
***Gait***	0-3	0.00 ± 0.00	0.60 ± 0.24	*0.0239
***Pelvic Elevation***	0-3	2.00 ± 0.00	1.30 ± 0.20	**0.0037
***Tail Elevation***	0-2	1.00 ± 0.00	0.30 ± 0.20	**0.0037
***Startle Response***	0-3	0.58 ± 0.15	0.60 ± 0.10	0.9329
***Touch Escape***	0-3	0.17 ± 0.11	0.70 ± 0.12	**0.0089
***Trunk Curl***	0-1	0.00 ± 0.00	0.00 ± 0.00	N.A.
***Limb Clasp***	0-1	0.00 ± 0.00	0.90 ± 0.10	**** < 0.0001
***Visual Placing***	0-4	2.25 ± 0.28	2.00 ± 0.16	0.4837
***Grip Strength***	0-4	1.33 ± 0.17	1.10 ± 0.10	0.2848
***Righting Reflex***	0-3	0.00 ± 0.00	0.50 ± 0.22	*0.0352
***Catalepsy (-ve Geotaxis)***	0-4	0.42 ± 0.27	0.50 ± 0.50	0.8812
***Air puff***	0-3	0.83 ± 0.17	0.70 ± 0.12	0.5503
***Fear***	0-1	0.00 ± 0.00	0.00 ± 0.00	N.A.
***Irritability***	0-1	0.00 ± 0.00	0.00 ± 0.00	N.A.
***Aggression***	0-1	0.00 ± 0.00	0.00 ± 0.00	N.A.
***Vocalization***	0-1	0.00 ± 0.00	0.00 ± 0.00	N.A.
***Confinement (30 s)***	0-3	0.00 ± 0.00	0.00 ± 0.00	N.A.
***Grip metal bar***	0-1	0.42 ± 0.20	0.60 ± 0.24	0.5727

Data are represented as the mean ± SEM. *n* = 5. Statistical analysis by unpaired 2-tailed T-Student test. *P < 0.05, **P < 0.01 and ****P < 0.0001 significantly different from WT.

### Young adult and aged gbCNP^–/–^ mice had increased blood brain barrier extravasation

**FIGURE 6:**
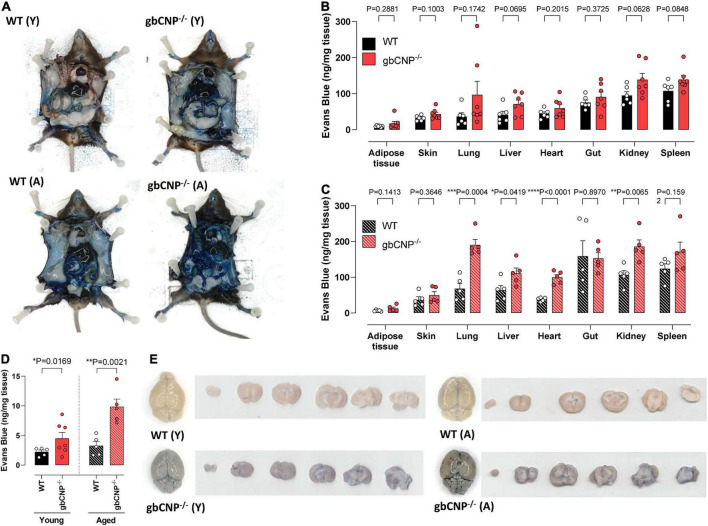
Young adult and aged gbCNP^– /–^ mice had increased blood brain barrier extravasation. Representative images of wild-type (WT) and global CNP knockout (gbCNP^– /–^) mice at 45 min following Evans blue intravenous administration **(A)**. Evans blue extravasation in the organs of young adult (12 weeks, Y; **B)** and aged (65 weeks, **A**,**C)** mice, and in the brain of WT and gbCNP^– /–^ animals **(D)**. Representative images of Evans blue extravasation in whole brains and brain sections from young adult (12 weeks, Y) and aged (65 weeks, A) mice **(E)**. *n* = 5-7. Statistical analysis by two-tailed Student’s t-test. Each statistical comparison undertaken has an assigned *P* value (adjusted for multiplicity).

Young adult gbCNP^–/–^ animals showed a trend to increased permeability in lung, liver, kidney and spleen but this failed to reach statistical significance (Figures 6A,B). However, there was a clear genotype difference observed in aged mice, especially in the case of the lung, liver, heart, and kidney (Figures 6A,C). Importantly, both young adult and aged gbCNP^–/–^ mice had increased vascular permeability in the brain (Figures 6D,E). These data imply that the BBB is disrupted in gbCNP^–/–^ mice from an early age and worsens with time.

### CNP prevented endothelial cell extravasation via NPR-C signaling

**FIGURE 7:**
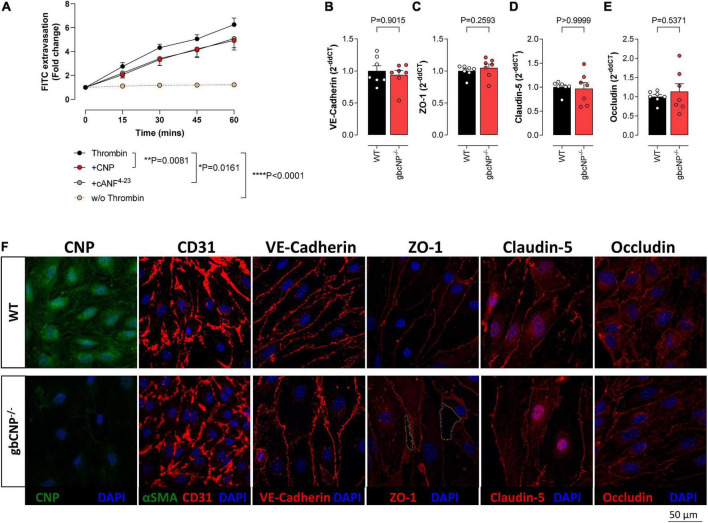
CNP prevented endothelial cell extravasation via NPR-C signaling. Transwell FITC extravasation from murine brain endothelial cells from wild-type (WT) animals induced by thrombin (5 IU/ml), in the absence and presence of CNP (100 nM) or the NPR-C selective agonist cANF^4–23^ (100 nM), compared to basal unstimulated extravasation (w/o Thrombin); **(A)**. mRNA expression of VE-cadherin **(B)**, Zonula Occludens-1 (ZO-1); **(C)**, claudin-5 (Cldn5); **(D)** and occludin **(E)**, and representative images of C-type natriuretic peptide (CNP), CD31, VE-cadherin, ZO-1, claudin-5 and occludin inmmunohistofluorescence in murine brain endothelial cells from wild-type (WT) and global CNP knockout (gbCNP^–/–^) mice **(F)**. The white dotted line indicates a gap between the cells. *n* = 5-7. Statistical analysis by two-tailed Student’s *t*-test, Mann–Whitney *U* test or two-way analysis of variance with Šídák *post hoc* test. Each statistical comparison undertaken has an assigned *P* value (adjusted for multiplicity).

To confirm which cognate receptor(s) mediates the effect of CNP deletion on endothelial integrity, an extravasation assay was performed in murine brain endothelial cells from WT animals. FITC extravasation from untreated cells was minimal. However, when challenged with thrombin, a potent endothelial permeability disruptor (Rabiet et al., 1996), the dye extravasation increased steadily for the duration of the assay (Figure 7A). Both CNP and the NPR-C selective agonist cANF^4–23^ were able to partially inhibit this effect (Figure 7A). Next, we evaluated the expression of tight junction proteins in whole brain homogenates of WT and gbCNP^–/–^ mice. mRNA expression of adherens junction protein (VE-cadherin) and tight junction proteins (ZO-1, occludin, and claudin-5) was not altered in gbCNP^–/–^ mice compared to their WT littermates (Figures 7B-E). Next, we studied the spatial expression of these proteins in WT and gbCNP^–/–^ endothelial cells isolated from the brain. Endothelial cell purity was assessed by sequential staining with CD31 (endothelial cell specific marker) and α-SMA positive cells (fibroblast, pericytes, smooth muscle cells). The purity of the cultures was over 99% both in WT and gbCNP^–/–^ cultures, with only a few isolated α-SMA positive cells. CNP expression was significantly reduced in cells isolated from gbCNP^–/–^ mice (Figure 7F). Next, we evaluated the expression of adherens and tight junction proteins. The levels of mRNA expression and the protein distribution along the cell membranes of VE-cadherin, claudin-5 and occludin were similar between WT and transgenic mice (Figures 7B-F). However, although overall mRNA expression of ZO-1 was unaltered (Figure 7C), gbCNP^–/–^ animals exhibited a disorganized, juxta-membrane distribution of ZO-1, revealing gaps between the cells (Figure 7F).

## Discussion

A central role for CNP has been established in relation to its NPR-B/cGMP-mediated promotion of axonal bifurcation in somatosensory neurons and auditory function (Dumoulin et al., 2021; Marchetta et al., 2022). However, a wider contribution of the peptide to the physiological regulation of cognition, motor function and BBB integrity has not been fully elucidated, despite previous work hinting at a role for CNP in these settings (Vigne and Frelin, 1992; Pedram et al., 2002; Barmashenko et al., 2014; Rapley et al., 2018). Herein, we utilized a mouse model with inducible, global deletion of CNP, which allowed us to demonstrate the detrimental effects of decreased CNP levels on spontaneous locomotor activity, sensorimotor function, gait coordination and vascular permeability.

Previously, we reported that gbCNP^–/–^ mice have similar levels of spontaneous activity compared to WT mice (Perez-Ternero et al., 2022). However, these results were acquired in the home cage, an environment to which the mice were habituated. Herein, gait and coordination also showed no difference between genotypes after the animals had been familiarized with the testing apparatus (e.g., CatWalk*™*). In contrast, gbCNP^–/–^ animals showed increased locomotor activity when exposed to novel environments (the open field and elevated zero maze); moreover, genetic deletion of CNP resulted in diminished performance with increasing speed of the rotarod task. These observations indicate that gbCNP^–/–^ animals are more active but perform worse when they are exposed to novel or more challenging situations. Reduced performance on the rotarod, beam crossing and stick suspension might be explained by altered motor coordination or balance; however, gbCNP^–/–^ mice did not show any motor impairment on flat ground, or in the open field and zero maze. Thus, the lower performance on motor coordination tests is not related to a physical inability to complete the test, but rather an unwillingness to perform more demanding tasks (e.g., rotarod or crossing narrow beams). These effects of CNP deletion might also be underpinned by changes in motor learning and exploratory behavior. Previous studies have demonstrated that impaired neuron branching following disruption of CNP/NPR-B signaling does not affect locomotor activity (Tröster et al., 2018; Dumoulin et al., 2021), but results in enhanced exploratory and learning behavior (Barmashenko et al., 2014). Moreover, CNP levels in the brain change after environmental enrichment (Telegdy et al., 1999; Rapley et al., 2018).

Published data indicate that CNP administration can also affect anxiety, but with contradictory findings (Bíró et al., 1996; Montkowski et al., 1998). Anxiolytic actions of CNP have been shown to be dependent on dopamine receptor and/or α- or β- adrenoreceptor activation, or possibly via CNP-triggered suppression of plasma cortisol levels (Charles et al., 1992). In contrast, anxiogenic effects of the peptide have been reported that are mediated via enhancement of the corticotropin-releasing hormone (CRH)-induced release of cortisol (Kellner et al., 1997); a parallel anxiogenic action of CNP is also observed in healthy human volunteers (Yue et al., 2014). Indeed, CNP gene expression in the CNS is altered in various stress-related regions of the brain following chronic treatment with corticosterone in an *in vivo* model of depression (Li et al., 2019). However, in the present study, differences in anxiety were not detected; this may be explained by the testing conditions being only mildly stressful and any potential anxiogenic phenotype might have been confounded by the higher activity in gbCNP^–/–^ mice (i.e., diminished grooming would result from higher activity rather than changes in anxiety).

Since the altered behavioral profile, including the development of seizures, observed in gbCNP^–/–^ mice mimicked many of the consequences of BBB disruption (Gorter et al., 2015), we explored endothelial integrity in animals lacking CNP. Vascular permeability is increased in gbCNP^–/–^ mice, not only in their brain but also in other organs. Moreover, we show that the effects of CNP on vascular permeability are governed predominantly by NPR-C signaling since CNP and the NPR-C agonist cANF^4–23^ were equipotent in inhibiting thrombin-induced albumin extravasation. ANP, CNP and cANF^4–23^ have been reported previously to inhibit VEGF-mediated vascular permeability that is dependent on architectural disorganization of the cytoskeleton involving the tight junction protein ZO-1, which is critical for intercellular junctions, cell-cell tension, angiogenesis and endothelial barrier formation (Tornavaca et al., 2015). This action is achieved synergistically via signaling through NPR-A, NPR-B, and NPR-C, which regulate downstream pathways including Src, ERK1/2, JNK, and phosphatidylinositol 3-kinase/AKT (Vigne and Frelin, 1992; Pedram et al., 2002). Despite the fact that mRNA expression of tight junction proteins remained unchanged, murine brain endothelial cells from gbCNP^–/–^ mice revealed a similar disruption of ZO-1 distribution, intimating a common mechanism. Accordingly, CNP has been shown to protect BBB integrity following ischemic injury in mice overexpressing CNP (Ma and Zhang, 2018) and promote endothelial cell proliferation and angiogenesis (Bubb et al., 2019); indeed, endothelial cells express both NPR-B and NPR-C (Ma and Zhang, 2018; Bubb et al., 2019). In addition to tight junctions, endothelial cells control permeability via cytoskeleton components, such as actin filaments and microtubules, and such mechanisms may also underlie the protective effects of CNP. A good example being GEF-H1, a microtubule-associated RhoA-activating factor, that interacts with NPR-C (Nishida et al., 2021). Upon NPR-C activation, GEF-H1 increases endothelial permeability via enhanced exocytosis and cytoskeletal modulation (Birukova et al., 2006; Pathak and Dermardirossian, 2013; Tian et al., 2014), perhaps explaining the BBB disruption in gbCNP^–/–^ animals. Yet, natriuretic peptides, including CNP, have also been reported to increase vascular permeability via cGMP (Sarker and Fraser, 2002), downregulation of ZO-1 (Bohara et al., 2014) and changes to the endothelial glycocalyx (Jacob et al., 2013). These actions, however, appear to require higher concentrations of the peptides in a pathophysiological context (e.g., sepsis or heart failure).

A further link between BBB permeability, seizures and CNP is provided by the observations that NT-proCNP levels are reduced in patients with epilepsy (van Vliet et al., 2007; Ceylan et al., 2018); whether the BBB disruption in gbCNP^–/–^ mice is the sole cause of the spontaneous seizures (or a consequence of the seizures) in these mice will require further investigation. However, this seems unlikely, as we did not observe parallel behavioral deficits in global NPR-C^–/–^ mice, suggesting that NPR-B also makes an important contribution to this phenomenon. Since both NPR-B and NPR-C are highly expressed on neurons and glia, a composite action of CNP on BBB integrity and neuronal function appears more plausible. Indeed, neurotransmitter imbalance and neuron hyperexcitability can result in seizures (Devinsky et al., 2018) and CNP has been shown to reduce neuron excitability in the absence of gamma-aminobutyric acid (GABA)_*A*_-mediated inhibition, suggesting that CNP downregulation (as in gbCNP^–/–^ mice) triggers higher neuronal excitability. Indeed, CNP inhibits both purinergic and adrenergic neurotransmission in peripheral tissue in a cGMP-independent manner, suggesting that these effects are NPR-C mediated, via the inhibition of neurotransmitter release (Trachte and Drewett, 1994). In addition to its neuromodulator activities, CNP has been shown to have neuroprotective effects and to enhance dopaminergic differentiation and maturation of neurons (Giovannini et al., 2021). These effects could act synergistically with the modulation of neurotransmission to protect CNS integrity and prevent seizures in advanced age. Neuronal hyperexcitability and lower neuronal plasticity and repair might, therefore, contribute to the spontaneous seizures observed in gbCNP^–/–^ mice in the present study (Lignani et al., 2020). As such, the development of a neuron-restricted CNP knockout mouse line would be very informative.

In conclusion, this study suggests a role for CNP in the regulation of BBB integrity, the modulation of locomotor reactivity to novel environments, and protection from spontaneous seizures. These findings contribute to our understanding of the importance of BBB integrity in age-related neurological disorders and in pathological conditions in which increased vascular permeability is a key element of the disease. Further studies are warranted to analyze the role of CNP in cognitive behavior, regulation of neuronal excitability and vascular permeability in more mechanistic detail, and whether targeting CNP signalling pharmacologically may represent a novel means of treating such disorders.

## Data availability statement

The original contributions presented in this study are included in the article/Supplementary material, further inquiries can be directed to the corresponding author.

## Ethics statement

All animal studies were carried out in accordance with the UK Animals (Scientific Procedures) Act of 1986 and were approved by a local Animal Welfare and Ethical Review Body.

## Author contributions

CP-T, PNP, CF, ATM-T, and AJH conceptualized and designed the study. CP-T, PNP, and CF performed experiments. JLT and AD provided scientific advice. CP-T, PNP, and AJH performed data analyses and figures preparation. All authors contributed to the manuscript preparation.

## Footnotes

1
https://www.dropbox.com/s/4qvh2xa4sf1o2rs/Perez-Ternero%20et%20al%202022%20J%20Neurosci.mp4?dl=0
