# Supplementary material for: C-type natriuretic peptide preserves central neurological function by maintaining blood-brain barrier integrity
Source: Front Mol Neurosci. 2022 Oct 4;15:991112. doi: 10.3389/fnmol.2022.991112 (PMC9577671; doi:10.3389/fnmol.2022.991112)
Supplement: Supplementary file 1 [file Data_Sheet_1.PDF]

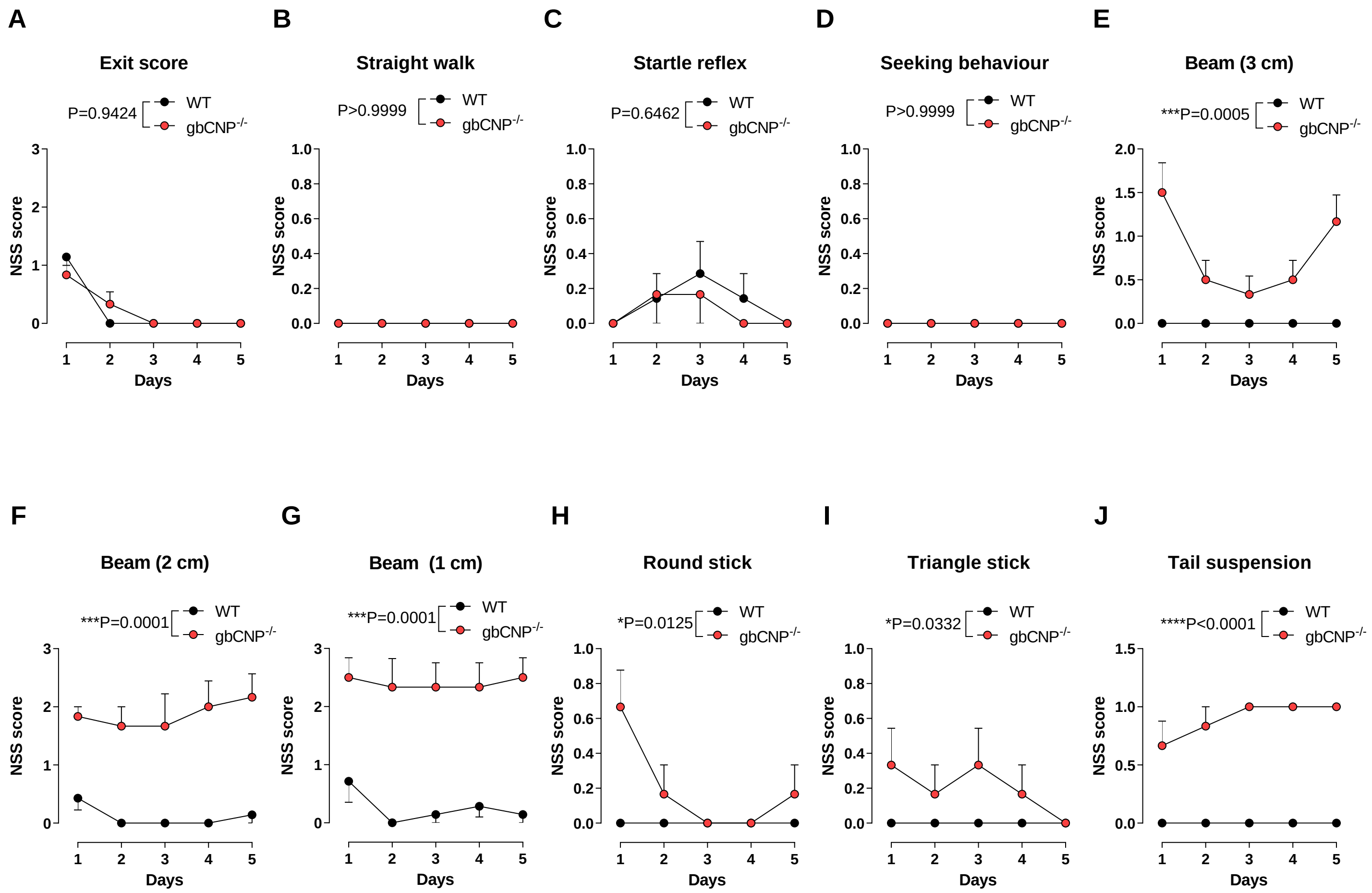

Supplementary Figure 1: Modified neurological severity score in wild-type and global CNP knockout mice.
Exit score (**A**), straight walking (**B**), startle reflex (**C**), seeking behavior (**D**), beam walking – 3 cm (**E**), beam walking – 2 cm (**F**), beam walking – 1 cm (**G**), round stick balance (**H**), triangle stick balance (**I**), and tail suspension (**J**) in wild-type (WT) and global CNP knockout (gbCNP$^{-/-}$) mice. n=6-7. Statistical analysis by two-way analysis of variance with Šídák *post-hoc* test. Each statistical comparison undertaken has an assigned *P* value (adjusted for multiplicity).

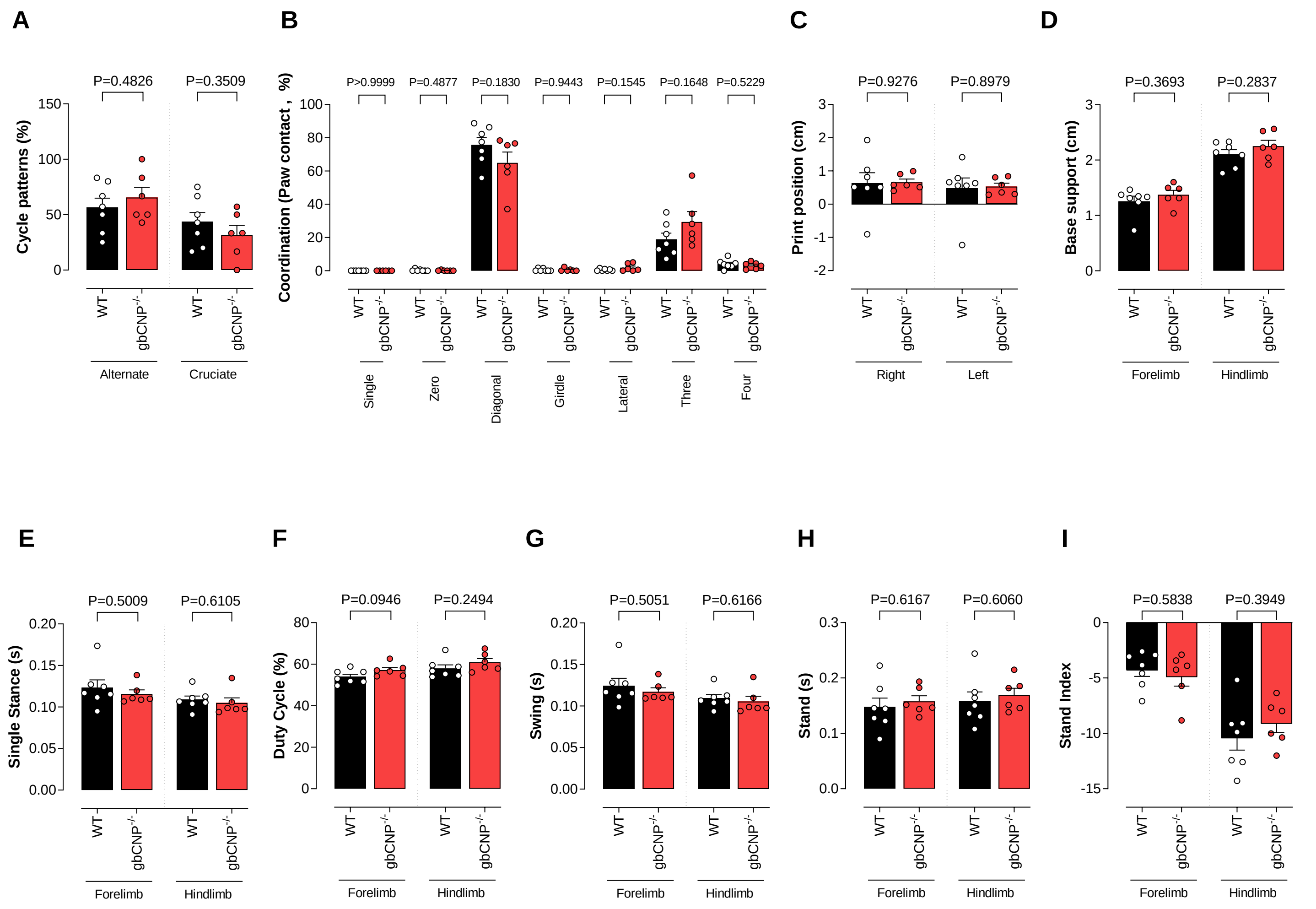

Supplementary Figure 2: CatWalk analyses of gait and coordination in wild-type and global CNP knockout mice

Cycle patterns (**A**), coordination (**B**), print position (**C**), base support (**D**), single stance (**E**), duty cycle (**F**), swing (**G**), stand (**H**) and stand index (**I**) in wild-type (WT) and global CNP knockout (gbCNP$^{-/-}$) mice. n=6-7. Statistical analysis by two-tailed Student's t-test. Each statistical comparison undertaken has an assigned *P* value (adjusted for multiplicity).
